# Development of a Population Pharmacokinetic Model Characterizing the Tissue Distribution of Resveratrol After Administration by Different Routes and Doses in Rats

**DOI:** 10.3390/nu17010181

**Published:** 2025-01-03

**Authors:** Cássia Cerqueira, Valdeene Santos, Jackeline Araújo, Laiz Pereira, Fabiana Batista, Denis Soares, Francine Azeredo, Ederlan Ferreira

**Affiliations:** 1 School of Pharmacy, Federal University of Bahia, Barão de Jeremoabo Street, Salvador 40170-115, Brazil; cassia.cerqueira90@hotmail.com (C.C.); enevieira@hotmail.com (V.S.); jackeline.marley20@gmail.com (J.A.); laiz.campos08@gmail.com (L.P.); fabianaprb@gmail.com (F.B.); denisms@ufba.br (D.S.); 2 Center for Pharmacometrics & System Pharmacology, College of Pharmacy, University of Florida, Orlando, FL 32610, USA; francinej@gmail.com

**Keywords:** pre-clinical study, method validation, HPLC/UV, pharmacokinetics parameters, blood–brain barrier

## Abstract

**Background:** Studies have demonstrated that resveratrol exerts several pharmacological effects. However, the pharmacokinetic parameters are not completely established. **Objectives:** This study describes the plasma pharmacokinetics and tissue distribution of resveratrol after administration by different routes and doses in rats. **Methods:** A reliable, simple, and sensitive HPLC method using UV detection for the quantification of resveratrol in rat plasma and tissues was developed and validated. In addition, a pharmacokinetic analysis using non-compartmental and population modeling was performed. **Results:** The pharmacokinetic parameters of resveratrol after the administration of 5 mg/kg via i.v. bolus calculated by non-compartmental analysis were a constant of elimination (ke) of 0.09 h^−1^ ± 0.04, a half-life (t1/2) of 9.5 h ± 3.7, an apparent volume of distribution (Vd) of 5.8 L/kg ± 4.7, a clearance (Cl) of 0.39 L/h/Kg ± 0.26, and an area under the curve (AUC) of 6076 ng/h/mL ± 2959. The results obtained after the administration of 100 mg/kg p.o. were an elimination constant (ke) of 0.12 ± 0.07 h^−1^, a half-life (t1/2) of 7.9 ± 4.2 h, the apparent volume distribution (Vd) of 13.3 ± 3.3 L/kg, a clearance (Cl) of 1.76 ± 0.49 L/h/Kg ± 0.26, and an area under the curve (AUC) of 6519 ± 1592 ng/h/mL. For the tissue distribution analysis, 10 mg/kg of resveratrol was intravenously administered to rats and the molecule was quantified in the liver, lung, kidney, heart, stomach, spleen, adipose tissue, and brain of the animals. **Conclusions:** The population pharmacokinetic modeling showed that resveratrol has a two-compartment model in both routes of administration and has a higher volume of distribution when it is given orally. In addition, resveratrol showed a high brain concentration after iv administration, which indicates that this molecule is capable of crossing the blood–brain barrier of animals, a crucial capacity for its neuroprotective activity.

## 1. Introduction

The 3,5,4 trihydroxy-trans-stilbene (resveratrol) is a polyphenolic antioxidant compound produced by a wide variety of plants including grapes, peanuts, blueberries, and blackberries, with red wine being the most widespread and known source [1,2]. Many studies have demonstrated several pharmacological effects exerted by resveratrol [3], mainly related to cardiovascular protection [4], neuroprotection [5], gut microbiota modulation, inflammatory disorders, and anticancer [6].

In addition to these, other beneficial actions of this compound have been reported, such as anti-hyperlipidemic, anti-aging, wound healing, and fighting infections [7].

Resveratrol is absorbed in the intestine by passive diffusion or by forming complexes with proteins present in the cell membrane, as to integrins. Once in the bloodstream, this compound can be found in three main forms: glucuronide, sulfate, or free. The free form of resveratrol can bind to albumin and lipoproteins, such as LDLs (low-density lipoproteins). However, since these complexes are dissociated in cell membranes that have receptors for albumin and LDL it allows resveratrol to be absorbed into cells [8]. The chemical structure of resveratrol indicates unfavorable pharmacokinetic properties, due to its low bioavailability, as it is rapidly and extensively metabolized and excreted. On the other hand, some studies have reconsidered this fact [9].

In this view, the distribution of resveratrol in target organs, such as the liver, can occur by converting the sulfates and glucuronides (metabolites) back to the compound [10]. In addition, another hypothesis suggests that the metabolites of resveratrol are easily reabsorbed after they undergo hydrolysis to the free form in the small intestine portion [11]. Although resveratrol is rapidly absorbed, its levels remain low due to its rapid metabolism [8].

Previous studies have established some pharmacokinetic parameters for resveratrol. The results of a clinical study showed that oral administration, in a single dose of 5.000 mg/resveratrol, reached the maximum plasma concentration of 2.4 µM of the compound after 30 min. In this same condition, the observed half-life was 1–3 h. However, when administering resveratrol in multiple doses, this half-life ranged between 2 and 5 h. In addition, the maximum observed concentration was 4.24 µM when the same dose was administered over 29 days [12].

Despite its potential for therapeutic, resveratrol has pharmaceutical limitations, such as the low bioavailability and pharmacokinetic parameters of compounds that are not completely established [13]. In this sense, the present study described plasma pharmacokinetics and tissue distribution of resveratrol after administration using different routes and doses in Wistar rats. Also, a population pharmacokinetics modeling approach was performed to better estimate all the pharmacokinetic parameters and micro-constants of resveratrol. Therefore, these results contribute to understanding the delivery system, bioavailability, and biological efficacy of resveratrol.

## 2. Materials and Methods

### 2.1. Reagents and Standards

Resveratrol (purity > 98%) was purchased from “Bothânica Manipulação e Homeopatia” (Araraquara, SP, Brazil). Acetonitrile and Milli-Q water were of HPLC grade. All other reagents used in the experiments were purchased from Sigma-Aldrich^®^ (Sigma, St Louis, MO, USA) when there is no specification.

### 2.2. Animals, Resveratrol Administration, and Sampling

The animals, 2- to 3-month-old male Wistar rats (250–300 g), prevenient from the Health Sciences Institute of UFBA Vivarium, were kept under control for 12 h of a light–dark cycle during the acclimatization period with ad libitum access to water and food. The resveratrol solution (3 mg/mL) was prepared with the addition of 10% DMSO, making up the final volume with saline. For the pharmacokinetic analysis, resveratrol was administered via the caudal lateral vein at a dose of 5 mg/kg (*n* = 6) and by oral gavage at a dose of 100 mg/kg (*n* = 6). For the tissue distribution study, resveratrol was administered intravenously at a 10 mg/kg dose (*n* = 12). The total number of rats used in this study was 24. The distribution of rats into groups for tail vein injection or gavage was performed randomly to minimize bias and ensure comparability between groups. Randomization was applied without specific stratification criteria, as all animals were of similar baseline characteristics (e.g., weight, age, and health status). All animal experiments were conducted in accordance with the Declaration of Helsinki and approved by the institutional ethics committee of the Federal University of Bahia prior to the study (protocol code 43/2016).

Aliquots of 10 µL of each sample (calibration and quality control) were added to 90 µL of blank rat plasma. After vortexing for 20 s, they were centrifuged (6000× *g*/10 min./4 °C). Then, cold methanol was added (ratio 3:1 *v*/*v*), mixed, and centrifuged (10,000× *g*/4 min./4 °C) to extract the biological matrices before injection.

### 2.3. Analysis of Resveratrol in Rat Biological Samples

Resveratrol solution (50 µg/mL) was prepared in methanol (70%). Standard calibration solutions (62.5, 250, 1000, 2000, and 5000 ηg/mL) and three quality control solutions (125, 500, and 2500 ηg/mL) were prepared by dilutions. All samples were previously filtered, using 0.45 μm filters (Millipore^®^, Merck, Burlington, NJ, USA).

The concentrations of resveratrol in rat biological samples by chromatographic analysis on performed using an HPLC system^®^ (Flexar™, PerkinElmer, Waltham, MA, USA), equipped with an automatic oven, UV–visible detector, and binary pump. A reverse-phase column (C18 × 4.2 mm × 150 mm, 5 μm, Phenomenex, Torrance, CA, USA), coupled with a pre-column (C18 × 10 mm × 5 μm, Hypersil BDS, Thermo Scientific, Waltham, MA, USA) was used. The mobile phase used consisted of Milli-Q water (A) and acetonitrile grade HPLC (B), and the gradient was as follows: 8.0 min at 10% (B); 5.0 min at 70% (B); and 7.0 min at 10% (B) to return to the initial conditions. The detection wavelength was set to 310 nm at a column temperature of 50 °C and a flow rate of 1.0 mL/min. The injection volume was 10 μL. In the present study, the LC-UV method was developed and validated for rat plasma and tissue samples.

### 2.4. Validation

The validation procedures were performed according to the guidelines of the FDA [14], which is presented as a guide for the validation of bioanalytical methods, to determine selectivity, linearity, precision, accuracy, recovery, and stability of the analyte in the biological matrix. As briefly presented below, blank rat plasma and tissue homogenate were used to establish and validate a standard protocol.

For linearity, the calibration curves were performed by quantifying five different concentrations of resveratrol solution in the range of 62.5–5000 ƞg/mL added to rat plasma. The analyses were performed in triplicate on two consecutive days for inter-day analysis. The linearity of the calibration curves was based on the peak area ratio, and the function of the nominal concentration was evaluated by linear regression. To accept the data, the precision needed to have a deviation less than or equal to 15% and less than or equal to 20% concerning the concentration known for the LQL. The selectivity was determined by UV scanning by analyzing blank rat plasma and rat plasma samples containing resveratrol. The results were compared and investigated for interferences in the peak chromatographic regions of resveratrol. Accuracy was calculated by comparing the quantified concentration in the experimental samples and the known concentration of resveratrol. The data acceptance criteria included the coefficient of variation within a 15% deviation, except for the LQL, for which values outside the 20% range of the known value are not allowed. In the validation test, accuracy was assessed using the coefficient of variation between replicates of quality controls on the calibration curve with concentrations of 125, 500, and 2500 ƞg/mL Precision is further subdivided into intra- and inter-day. The intra-day was defined by repetition in a short period, with the results obtained under the same conditions of analysis. In contrast, the inter-day was defined on two consecutive days, obtaining the results under different conditions of analysis. Relative standard deviation (RSE) values greater than 20% for LQL and 15% for other concentration values are not admitted [14]. Resveratrol recovery was performed in three different concentrations (125, 500, and 2500 ƞg/mL) and determined by the peak area ratio between samples extracted with resveratrol and resveratrol solutions. The experiment was performed in triplicate and the results were expressed as % recovery. The stability of the analyte (biological matrix stability) was evaluated by long-term storage in freezing at −20 °C, the samples were stored for the period of 30, 60, and 90 days, thawed, and evaluated. The experiment was carried out in triplicate and the data obtained were expressed in % of stability, the samples were considered stable if the results were within the limits of the variability of 15%.

### 2.5. Pharmacokinetic Study

Blood samples were collected through the caudal vein at times 0.125, 0.25, 0.5, 1, 1.5, 2, 4, 8, and 24 h after intravenous administration and 0.25, 0.5, 0.75, 1, 1.5, 2, 4, 6, 10, and 24 h after oral administration.

For the pharmacokinetic study, the plasma concentrations versus time profiles were analyzed individually for each animal using the non-compartmental approach before all values of plasma concentration were modeled using a population pharmacokinetic (POPPK) approach. In the non-compartmental analysis, the maximum plasma concentration (Cmax) and the maximum concentration–time (Tmax) were obtained by visual inspection of the plasma concentration-time profiles after oral administration. Pharmacokinetic parameters such as elimination rate constant (ke), the area under the curve (AUC0-∞), clearance (CLtot), half-life (t1/2), the volume of distribution (Vd), and bioavailability (Fabs) were determined using classic equations [15]. The POPPK modeling was performed using the software Monolix^®^ version 2020R1 (http://www.lixoft.eu, accessed on 22 March 2021). All fixed and random effects were estimated by calculating the maximum likelihood estimate of the parameters without any approximation of the model (without linearization) using the algorithm to maximize the expectation of stochastic approximation combined with a Monte Carlo procedure [16].

Interindividual variability was measured assuming a log-normal distribution, and each animal’s pharmacokinetic parameter was calculated as below:

$$\theta i=\theta *e^{ƞ}$$

where θi is the parameter value of an individual animal i, θ is the typical value of the population parameter, and e is a random variable with mean zero and variance ω^2^.

Residual variability was described by the proportional error model; thus, concentration estimates for each time (j) and animal (i) were obtained using the equation:

Yij=Fij+Fij∗εij

where Yij is the observed concentration, Fij is the estimated concentration, and εij is a random variable with mean zero and variance σ^2^.

The likelihood ratio test, incorporating log-likelihood values, the Akaike information criterion (AIC), and the Bayesian information criterion (BIC), was used to evaluate different hypotheses regarding the final model and the residual variability model. From the final model, 1000 simulations were performed to calculate the visual predictive check (VPC) and normalized prediction distribution error (NPDE) metrics. The mean and variance of these metrics should ideally be close to 0 and 1, respectively, and their distributions should resemble a normal distribution.

### 2.6. Resveratrol: Tissue Distribution Assay

For the tissue distribution study, after an iv bolus administration of 10 mg/kg of resveratrol, three animals were euthanized by guillotine decapitation to recover chemically uncontaminated tissues at 1 h, 3 h, 6 h, and 11 h (*n* = 12). Samples of the liver, lung, kidney, heart, stomach, spleen, adipose tissue, and brain were removed, weighed, and frozen at −80 °C. Also, whole blood samples were collected in the tube (Eppendorf^®^, Hamburg, Germany) previously heparinized tubes. The tissue samples were left to thaw and weighed, and 2 mL of methanol was added per g of tissue, homogenized, and centrifuged (3500× *g*/15 min./4 °C). The supernatant was processed similarly to the plasma samples and analyzed using the HPLC-UV method previously developed and validated.

### 2.7. Resveratrol: Plasma Protein Binding Assay

The rat plasma protein binding of resveratrol was determined by the ultrafiltration technique. Aliquots of 100 mL of resveratrol solutions at different concentrations were added to 900 mL of rat plasma to give final concentrations of 0.5, 1.0, 10.0, and 50.0 µg/mL. After mixing for 30 s in a vortex shaker, 100 mL of these samples were used to determine the total concentration of the substance. Samples were processed and analyzed as described in the HPLC-UV methodology described. For determining the concentration of unbound resveratrol, the remaining volume (900 mL) was added to Centrifree (Millipore^®^, MA, USA) filtration tubes and centrifuged (2000× *g*/20 min. at 21 °C). Aliquots of 100 mL of the filtrate were then processed as described in the “Recovery” methodology section. The experiment was performed in triplicate. Resveratrol plasma protein binding (fu) was calculated from the unbound drug concentration (Cu) determined in the filtrate and the total plasma concentration (Ct) using the following equation:

$$fu=\frac{Cu}{Ct}$$

## 3. Results

### 3.1. Method Validation

In this study, a reliable, simple, and sensitive HPLC method using UV detection for the quantification of resveratrol in rat plasma and tissues was developed and validated. The validation procedures were performed according to the guidelines of the FDA, which is presented as a guide for the validation of bioanalytical methods to determine the selectivity, linearity, precision, accuracy, recovery, and stability of the analyte in the biological matrix [14].

The method was linear at the concentration range of 62.5–5000 ηg/mL in rat plasma (Figure S1a). The calibration curves were linear using weighted linear regression (1 = concentration), the mean equation of the validation calibration curve was: y = 44.285x + 2130.9, R^2^ = 1, where y represents the area of the resveratrol peak and x represents the plasma concentration of the analyte. Selectivity can be observed by comparing the rat plasma chromatograms containing resveratrol (Figure S1b) compared to the blank sample (Figure S1c). No absorption peaks were detected that could interfere with the resveratrol absorption peak in the UV spectrum at 310 nm, thus confirming the selectivity of the method. The precision results are shown in Table S1, agreeing with the recommendation by the FDA guide [14], where the CV must not exceed 15%, except the LIQ, where it must not exceed 20%. The relative intra and inter-day standard deviation for resveratrol during the validation experiment is shown in Table S2 (Supplementary Material). The accuracy of the bioanalytical method was determined between 85.01 and 115.03% for all quality control samples.

The recovery of the samples proved to be independent of the concentrations evaluated and was 20.1 ± 3.8%. Although low, the fact that recovery was not statistically different between the different concentrations evaluated indicates that the method of extraction of resveratrol in plasma is correct for a pharmacokinetic study. The stability in the biological matrix showed that the storage of resveratrol in the period of 30, 60, and 90 days showed acceptable variability within the limits of 15% of the known concentration (Table S3, Supplementary Material). As there is no statistical difference (α = 0.05) between the values, the results indicate that the samples can remain in the freezer (−20 °C) for up to 90 days to be analyzed by HPLC.

### 3.2. Pharmacokinetics Modeling

**Figure 1:**
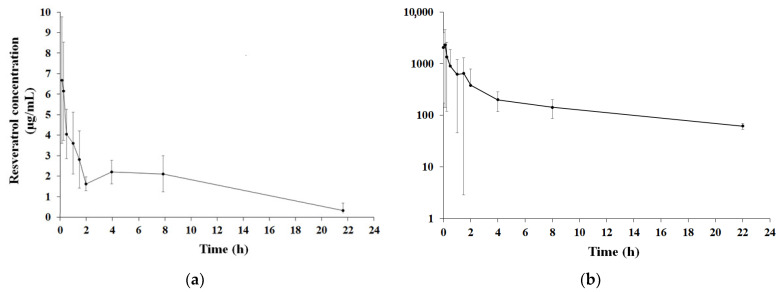
Plasma concentration profile of resveratrol after i.v. bolus (**a**) and oral (**b**) doses administration. The dose administered of 5 mg/kg and 100 mg/kg, respectively, in male Wistar rats (Mean ± SD, *n* = 6).

The mean concentration–time profile of resveratrol after 5 mg/kg of resveratrol was administered via iv bolus to Wistar rats (*n* = 6) can be seen in Figure 1a. The parameters calculated by the non-compartmental approach were a constant of elimination (Ke) of 0.09 h^−1^ ± 0.04, a half-life (t1/2) of 9.5 ± 3.7 h, the apparent volume of distribution (Vd) of 5.8 L/kg ± 4.7, a clearance (Cl) of 0.39 L/h/Kg ± 0.26, a total area under the curve (AUC) of 6076 ng/h/mL ± 2959, and an average residence time (MRT) of 8.7 h ± 3.4.

The average concentration curve of resveratrol after oral administration of 100 mg/kg dose is shown in Figure 1b. As can be seen, resveratrol has shown two concentration peaks at 2 and 6 h after its oral administration. These data indicates that the molecule can undergo an enterohepatic recirculation, staying longer in the organism, thus increasing its disposition in it. The parameters calculated by the non-compartmental approach were an elimination constant (Ke) of 0.12 ± 0.07 h^−1^, a half-life (t1/2) of 7.9 ± 4.2 h, an apparent volume distribution (Vd) of 23.3 ± 3.3 L/kg, a clearance (Cl) of 1.76 ± 0.49 L/h/Kg ± 0.26, a total area under the curve (AUC) of 6519 ± 1592 ng/h/mL, and a mean residence time (MRT) of 7.7 ± 1.7 h. The bioavailability (fabs) calculated using the equation described in Davit, Conner, and Shargel [17] was approximately 6%.

**Figure 2:**
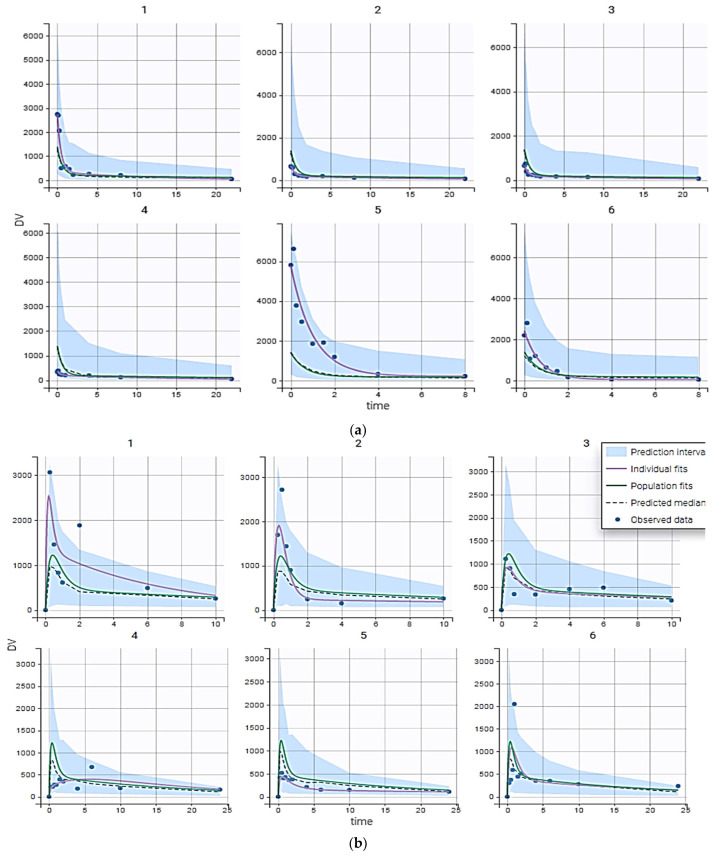
Individual time curves of resveratrol after i.v. bolus (**a**) and oral (**b**) administration in rats. Blue points, observations; purple line, typical population prediction; green line, individual prediction; hashed line, predicted median concentration; and blue areas, predictions with a 90% confidence interval.

The population pharmacokinetic modeling performed after the administration of resveratrol by oral and intravenous routes in dosages of 100 and 5 mg/kg, respectively, are shown in Figure 2.

An open two-compartment model was best fitted to the plasma concentrations of resveratrol after the iv administration in Wistar rats:

$$\frac{dC}{dt}=+k21*P-k12*C-k10*C$$

$$\frac{dP}{dt}=+k21*P-k12*C$$

where C is the total plasma concentration, P is the total peripheric concentration; k_10_ is the elimination rate, and k_12_ and k_21_ are the distribution and redistribution rates, respectively; and t is time.

An open model of two compartments with first-order elimination and absorption was best fitted to the plasma concentrations of resveratrol after oral administration in Wistar rats:

$$\frac{dA}{dt}=-ka*A$$

$$\frac{dC}{dt}=+ka.A+k21*P-k12*C-k10*C$$

$$\frac{dP}{dt}=+k21*P-k12*C$$

where C is the total plasma concentration, P is the total peripheric concentration, and A is the total amount of the drug in the absorption site; ka is the absorption rate, k_10_ is the elimination rate, and k_12_ and k_12_ are the distribution and redistribution rates, respectively; and t is time.

The values of population pharmacokinetic micro-constants and parameters, as the inter-individual variability values estimated, are shown in Table 1.

**Figure 3:**
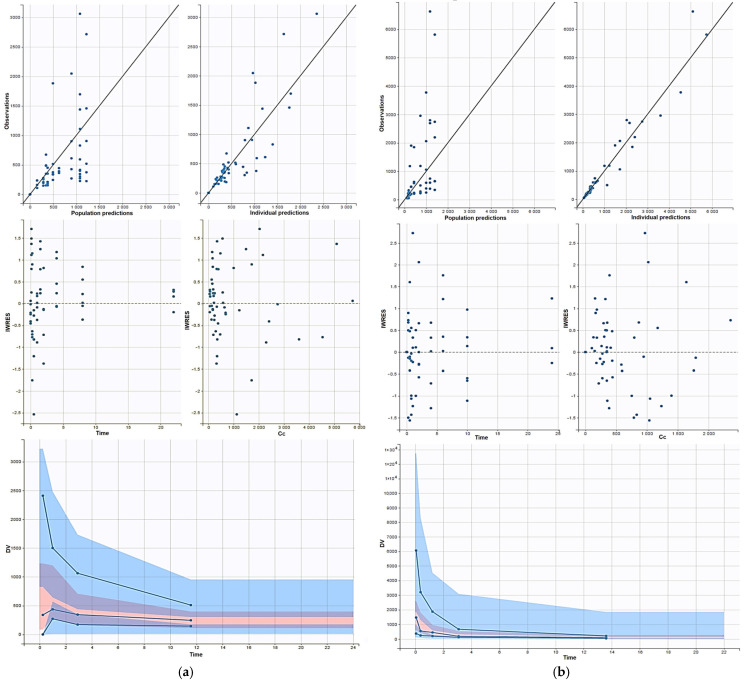
The goodness of fit plots of oral administration (**a**) and i.v. bolus administration (**b**). Correlation between observed plasma concentration and population plasma concentration (upper left panel) and individual predicted (upper right panel). Homogeneous distribution of residues about predicted plasma concentration and time (middle panel). Visual predicted check values (lower panel). The solid lines represent the 5th, 50th, and 95th percentiles of the observed data. The dotted lines represent the 5th, 50th, and 95th percentiles of the simulated data. The areas indicate the range of 90% predicted associated with the 5th, 50th, and 95th percentiles of the simulated data. The blue circles correspond to the data observed while the blacks to the simulated ones.

The goodness of fit plots of the population modeling are shown in Figure 3. It can be seen that both models were able to fit the experimental data well.

### 3.3. Tissue Distribution

**Figure 4:**
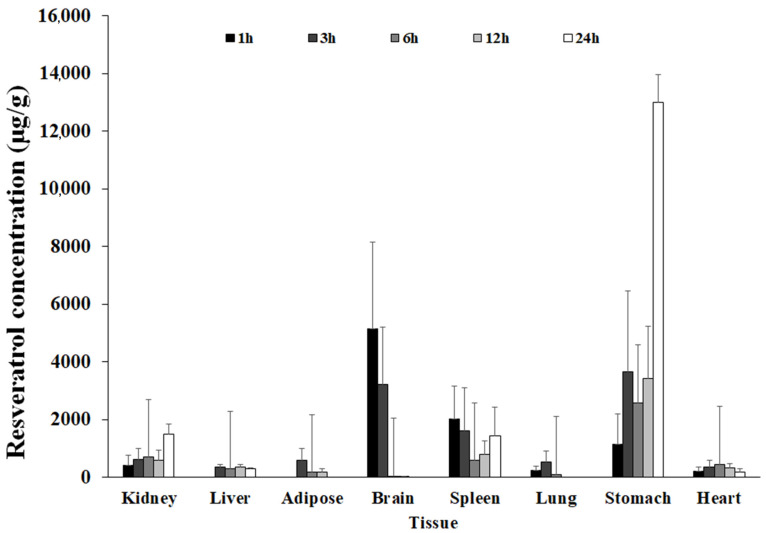
Resveratrol tissue distribution after administration. A dose administered of 10 mg/kg i.v to male Wistar rats (Mean ± SD, *n* = 3/time point).

The tissue concentration values of resveratrol over time in different organs and tissues can be seen below after administration of 10 mg/kg intravenous dose of resveratrol to Wistar rats (*n* = 3/point), as shown in Figure 4.

### 3.4. Plasma Protein Binding

The plasma protein binding of resveratrol in rats was determined using a concentration range of 0.5–50 µg/mL, which encompasses the range of concentrations observed in the plasma pharmacokinetics study. The amount of resveratrol bound to the ultrafiltration membrane was quantified, and this value was used to adjust the final protein binding value. The results indicated that approximately 79 ± 5% of the drug binds to plasma protein, and this binding was linear across the analyzed concentration range.

## 4. Discussion

In the present study, we described the plasma pharmacokinetics and tissue distribution of resveratrol after administration using different routes and doses in Wistar rats. For this, a reliable, simple, and sensitive HPLC method using UV detection for the quantification of resveratrol in rat plasma and tissues was validated.

The selectivity assessment results of the bioanalytical method demonstrate that the method developed to quantify resveratrol in rat plasma samples is suitable for pharmacokinetic studies. Chromatograms display distinct peaks, with the resveratrol peak distinguishable from endogenous peaks in both blank and spiked plasma samples. This indicates minimal interference from endogenous components. This underscores the method’s accuracy in measuring resveratrol concentrations in rat plasma [10,14].

The method demonstrates high sensitivity, with a limit of quantification (LLOQ) as low as 62.5 ng/mL, making it suitable for detecting minimal concentrations of resveratrol, an essential feature for preclinical pharmacokinetic studies where analyte levels may fluctuate [14]. Evaluation of precision and accuracy across a range of concentrations yielded results within acceptable limits. Mean values for quality control samples were typically within ±15% of target concentrations, except for the LLOQ, which fell within ±20%, underscoring the method’s robustness. Calibration curves confirmed linearity over a dynamic range of 62.5 to 5000 ng/mL, displaying strong correlation coefficients and minimal deviations from expected values, establishing the method as reliable for analyzing resveratrol in rat plasma samples [18,19,20].

The pharmacokinetic analysis of resveratrol after intravenous (IV) administration revealed substantial variability in key parameters, including elimination half-life (t 1/2), volume of distribution (Vd), and clearance (Cl), compared to prior studies [18,19,21]. These differences may arise from inter-animal variability, variations in experimental conditions [20], or analytical methods [21,22]. Additionally, interspecies differences in the metabolism of resveratrol, particularly in sulfation and glucuronidation pathways, may contribute to these observations. Specifically, Chiu and Huskey (1998) demonstrated species-dependent differences in UDP-glucuronosyltransferases (UGTs) activity, which could impact resveratrol’s clearance and bioavailability [23]. Similarly, Kutsukake et al. (2018) highlighted that Wistar rats exhibit higher glucuronidation rates compared to Sprague Dawley rats, potentially leading to lower systemic exposure [24]. Wang et al. (2021) demonstrated that while the in vitro intrinsic clearance results for several compounds showed few differences between Sprague Dawley and Wistar strains in rat hepatocytes, significant variability in metabolite formation rates was observed for certain drugs. These findings emphasize that differences in metabolic pathways and metabolite identity can be drug-dependent, potentially contributing to the pharmacokinetic variability in vivo [25]. The lower clearance observed in our study suggests potential metabolic impairment in the Wistar rats, as resveratrol undergoes extensive metabolism for elimination [9,10]. This finding contrasts with previous studies reporting relative clearance values, while our study provides total clearance, already accounting for resveratrol’s bioavailability. This highlights the importance of considering physiological factors such as hepatic enzyme activity, renal function, and strain-specific metabolic capacity, which play key roles in influencing drug total clearance [20,24,25]. Additionally, factors such as hepatic blood flow, tissue perfusion, and differences in transporter-mediated excretion could also impact systemic clearance and distribution [25]. Additionally, the use of different quantification techniques, such as LC-MS/MS in the compared studies, highlights the impact of methodological differences on pharmacokinetic outcomes [21].

The oral bioavailability of resveratrol in rats, estimated at approximately 6%, reflects its extensive first-pass metabolism, a common limitation for polyphenolic compounds like resveratrol [26,27]. This low bioavailability restricts the therapeutic potential of resveratrol when administered orally, despite its promising pharmacological properties. Similar challenges are observed in human studies, as highlighted by a meta-analysis of clinical trials that revealed substantial variability in pharmacokinetic parameters, including Cmax and Tmax, across different doses and studies. Notably, the meta-analysis identified a linear dose–response relationship for free resveratrol plasma concentrations, with medium doses (100–500 mg) achieving Cmax values comparable to the overall mean, suggesting an optimal dosing range with minimal risk of side effects [28]. The presence of dual concentration peaks in the plasma profile indicates enterohepatic recirculation, which prolongs systemic exposure and increases the molecule’s residence time. This phenomenon is further supported by the high apparent volume of distribution (Vd) observed in oral administration, reflecting extensive tissue distribution.

The population pharmacokinetics modeling approach was performed to better estimate all the pharmacokinetic parameters and micro-constants of resveratrol. The results corroborated these findings, with a two-compartment model incorporating first-order absorption and elimination providing a robust fit to oral pharmacokinetics. Our data underscore the challenges of optimizing oral bioavailability for resveratrol. The population pharmacokinetic modeling effectively described resveratrol’s pharmacokinetics for both IV and oral routes, with distinct models fitting the respective profiles. Goodness-of-fit plots demonstrated that both models adequately captured the experimental data, with minimal bias and well-distributed residuals across time. Moreover, the VPCs and relative standard error (RSE%) values for parameters and inter-individual variability were within acceptable limits (<30%), indicating model robustness and reliability [29]. These results validate the applicability of the chosen models for describing resveratrol pharmacokinetics and provide a foundation for further exploration of its disposition and therapeutic potential [18,30].

The tissue distribution profile of resveratrol provides valuable insights into its pharmacokinetics and potential therapeutic implications. The observed increase in resveratrol concentration in the stomach 11 h post-administration is notable and could suggest enterohepatic recirculation or delayed gastric clearance, mechanisms that may prolong the presence of resveratrol in the system [18]. This extended exposure could influence its therapeutic efficacy or toxicity, depending on the dose and target tissue [11]. The detection of resveratrol in the brain is particularly significant, as it confirms the molecule’s ability to cross the blood–brain barrier (BBB) [2,31,32,33].

In the study, the increased brain concentration of resveratrol after intravenous administration sheds new light on its neuroprotective potential. This property aligns with its proposed neuroprotective effects, supporting its potential use in treating or preventing neurological disorders [5]. However, the concentration and persistence of resveratrol in the brain should be further explored to better understand the extent and duration of its pharmacological action within the central nervous system. As expected, the rapid decline in resveratrol concentrations in the liver and kidneys after 3 h aligns with their primary roles in the metabolism and elimination of xenobiotics [8]. This observation underscores the importance of these organs in resveratrol’s clearance and raises considerations regarding potential accumulation or toxicity with repeated dosing. Overall, the tissue distribution data highlight key pharmacokinetic properties of resveratrol that are essential for optimizing its therapeutic applications and understanding its safety profile [13].

The plasma protein binding (PPB) of resveratrol in rats was 79 ± 5%, consistent with a high degree of protein association, which can significantly influence the drug’s pharmacokinetics and pharmacodynamics. The linearity of binding across the tested concentration range (0.5–50 µg/mL) suggests a lack of saturation of binding sites within this range, indicating that changes in plasma concentrations are unlikely to alter the fraction of unbound drug [34,35]. This behavior is critical for interpreting pharmacokinetic data, as the unbound fraction is pharmacologically active and available for distribution, metabolism, and elimination [36]. These findings are relevant to resveratrol’s disposition, as its high protein binding could contribute to its relatively low clearance and extended half-life observed in pharmacokinetic studies, particularly following intravenous administration. Understanding PPB is essential for extrapolating preclinical findings to clinical settings, especially when evaluating drug–drug interactions or disease-related changes in protein binding capacity [37]. The evidence presented in this present study contributes to understanding the delivery system, bioavailability, and biological efficacy of resveratrol. However, it is important to emphasize that these parameters must still be established for the metabolites derived from resveratrol.

## 5. Conclusions

An efficient and reliable HPLC/UV method for the quantification of resveratrol in rat plasma was developed and validated. This method proved to be sensitive, linear, precise, and accurate with success in its pharmacokinetic applicability in rats. The non-compartmental and population pharmacokinetic evaluation after oral and intravenous administration showed that resveratrol presented the ideal model of two compartments in both scenarios, with a longer exposure time in the body and greater distribution after oral administration when compared to intravenous administration, information validated by the population pharmacokinetic modeling. Resveratrol was quantified in the brain after iv administration, which indicates that this molecule is capable of crossing the blood–brain barrier of Wistar rats, which corroborates with its neuroprotective activity.

## Figures and Tables

**Table 1:** Estimated population pharmacokinetic parameters of resveratrol after administration.

PK Parameters and Microconstants Estimated	Mean i.v. (%RSE)	BSV i.v. (%RSE)	Mean Oral (%RSE)	BSV Oral (%RSE)
V (L/kg)	3.60 (19.9)	0.97 (29.4)	22.20 (29.5)	5.60 (13.6)
k_10_ (h^−1^)	0.17 (23.1)	0.07 (19.1)	0.43 (21.7)	0.09 (26.5)
k_12_ (h^−1^)	1.20 (17.4)	0.29 (24.6)	4.09 (23.1)	0.45 (22)
k_21_ (h^−1^)	0.26 (29.7)	0.11 (20.5)	0.44 (23.1)	0.33 (29.3)
ka (h^−1^)	-	-	1.88 (25.8)	0.49 (33)
Residual variability				
b	0.22 (11.8)	-	0.41 (12.3%)	-

Dose administered of 5 mg/kg intravenously and 100 mg/kg orally to Wistar rats (*n* = 6). %RSE, percentage of relative standard error; BSV, inter-individual variability (η).

## Data Availability

All methods and related data are presented in this paper. Additional inquiries should be addressed to the corresponding author.

## Supplementary Materials

The following supporting information can be downloaded at: https://www.mdpi.com/article/10.3390/nu17010181/s1, Figure S1: Resveratrol rat plasma calibration curve (a) and chromatographic profiles obtained from the plasma sample of rats with (b) and without (c) addition of resveratrol. Concentrations range from 625, 2500, 10,000, 20,000, and 50,000 ng/mL (Mean ± SD, *n* = 6); Table S1: Precision parameter of resveratrol in rat plasma; Table S2: Variation in the intra and inter-day accuracy of resveratrol in rat plasma; Table S3: Resveratrol stability in rat plasma.
