# Supplementary material for: Development of a Population Pharmacokinetic Model Characterizing the Tissue Distribution of Resveratrol After Administration by Different Routes and Doses in Rats
Source: Nutrients. 2025 Jan 3;17(1):181. doi: 10.3390/nu17010181 (PMC11722891; doi:10.3390/nu17010181)
Supplement: Supplementary file 1 [file nutrients-17-00181-s001.zip › nutrients-3369293-supplementary.pdf]

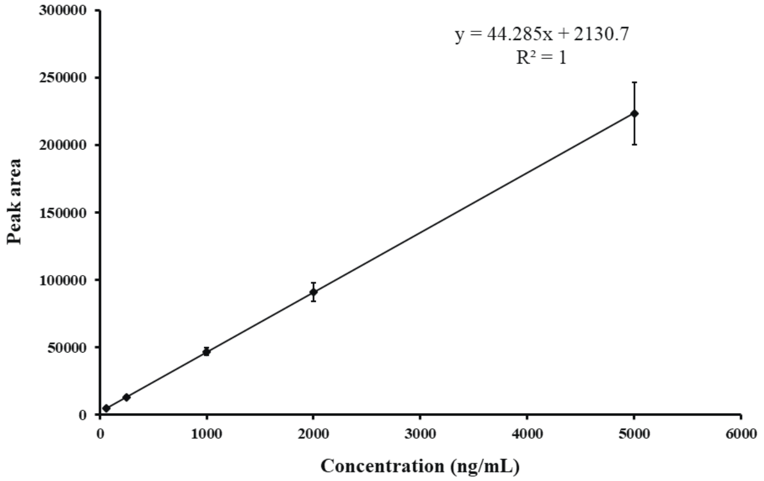

(**a**)

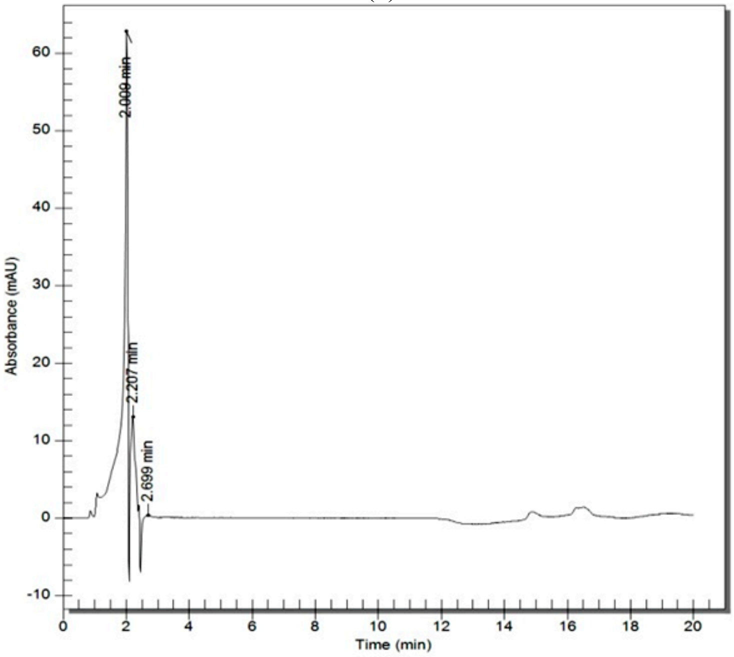

(**b**)

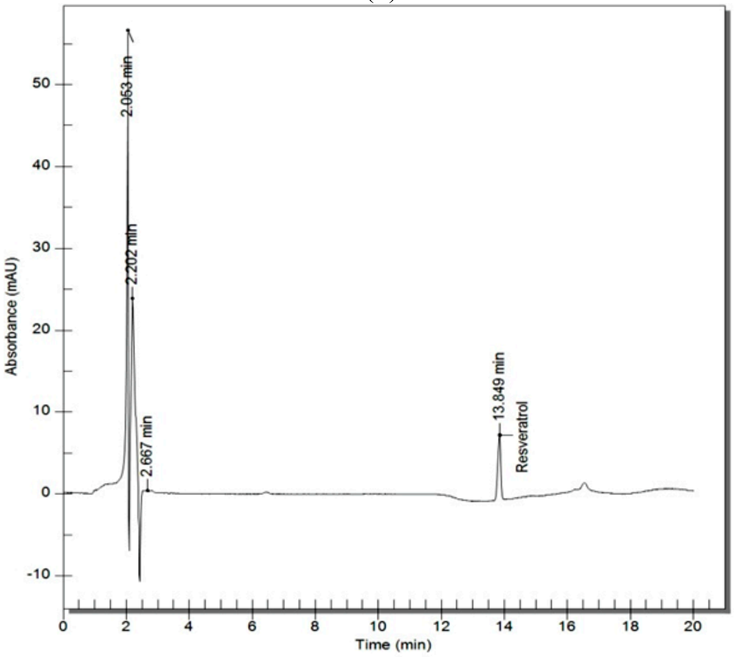

(**c**)

Figure S1. Resveratrol rat plasma calibration curve (**a**) and chromatographic profiles obtained from the plasma sample of rats with (**b**) and without (c) addition of resveratrol. Concentrations range from 625, 2.500, 10.000, 20.000, and 50.000 ng/mL (Mean ± SD, n = 6).

Table S1. Precision parameter of resveratrol in rat plasma.

Concentration of resveratrol (ng/mL)	CV% day 1	CV% day 2	CV% inter-day
62.5	17.68	11.41	19.30
250	2.51	10.22	2.75
1000	1.94	2.82	2.76
2000	1.64	1.90	1.09
5000	0.19	0.27	0.27

Dose administered 5 mg/kg intravenously and 100 mg/kg orally to Wistar rats (n = 6). %RSE, percentage of relative standard error; BSV, inter-individual variability (η).

Table S2. Variation in the intra and inter-day accuracy of resveratrol in rat plasma.

Concentration (ng/mL)	Quantified concentration			
	Day	Mean	*S.D.	**R.S.D. (%)
Intra-day				
125	1	117.85	13.64	11.57
	2	142.89	1.14	0.78
500	1	529.76	19.64	3.70
	2	531.44	14.34	2.69
2500	1	2696.12	14.35	5.32
	2	2267.45	10.18	4.49
Inter-day	-	139.95	29.41	2.10
	-	5385.42	70.96	1.32
	-	2528.81	2613.57	10.33

*SD, standard deviation. **R.S.S, Relative standard deviation

Table S3. Resveratrol stability in rat plasma.

Time (Days)	Stability (%)*
30	92.0 ± 6.6
60	95.6 ± 7.2
90	87.3 ± 3.2

*Concentration assessed of 125 and 2.500 ng/mL.
